# An institution-wide approach to submission, review, and funding of simulation-based curricula

**DOI:** 10.1186/s41077-017-0042-5

**Published:** 2017-06-15

**Authors:** David H. Salzman, Diane B. Wayne, Walter J. Eppich, Eric S. Hungness, Mark D. Adler, Christine S. Park, Katherine A. Barsness, William C. McGaghie, Jeffrey H. Barsuk

**Affiliations:** 1 Department of Emergency Medicine and Medical Education, Northwestern University Feinberg School of Medicine, 211 East Ontario Street, Suite 200, Chicago, IL 60611 USA; 2 Department of Medicine and Medical Education, Northwestern University Feinberg School of Medicine, 420 E. Superior St, 12th floor, Chicago, IL 60611 USA; 3 Ann & Robert H. Lurie Children’s Hospital of Chicago and Department of Medical Education, Northwestern University Feinberg School of Medicine, 240 East Huron St, McGaw 1-214, Chicago, IL 60611 USA; 4 Department of Surgery and Medical Education, Northwestern University Feinberg School of Medicine, 240 East Huron St, Chicago, IL 60611 USA; 5 Ann & Robert H. Lurie Children’s Hospital of Chicago and Department of Medical Education, Northwestern University Feinberg School of Medicine, 240 East Huron, McGaw 1-245, Chicago, IL 60611 USA; 6 Department of Anesthesiology, Northwestern University Feinberg School of Medicine, 251 E Huron St, Suite 5-704, Feinberg Pavilion, Chicago, IL 60611 USA; 7 Ann & Robert H. Lurie Children’s Hospital of Chicago and Department of Surgery, Northwestern University Feinberg School of Medicine, 225 East Chicago Ave, Chicago, IL 60611 USA; 8 Department of Medical Education, Northwestern University Feinberg School of Medicine, 240 East Huron St, McGaw 1-211, Chicago, IL 60611 USA; 9 Department of Medicine and Medical Education, Northwestern University Feinberg School of Medicine, 240 East Huron St, McGaw 1-236, Chicago, IL 60611 USA

**Keywords:** Medical curriculum, Medical simulation, Medical education, Proposal screening, Quality improvement

## Abstract

This article describes the development, implementation, and modification of an institutional process to evaluate and fund graduate medical education simulation curricula. The goals of this activity were to (a) establish a standardized mechanism for proposal submission and evaluation, (b) identify simulation-based medical education (SBME) curricula that would benefit from mentored improvement before implementation, and (c) ensure that funding decisions were fair and defensible. Our intent was to develop a process that was grounded in sound educational principles, allowed for efficient administrative oversight, ensured approved courses were high quality, encouraged simulation education research and scholarship, and provided opportunities for medical specialties that had not previously used SBME to receive mentoring and faculty development.

## Background

The recent expansion and popularity of simulation-based medical education (SBME) [1] has rapidly increased the demand for simulation space and resources [2]. SBME has also become a requirement or recommendation of various US Accreditation Council for Graduate Medical Education (ACGME) residency review committees and subspecialty boards [3–5]. This increasing interest in SBME and the need to meet professional regulatory requirements often coexists with finite time, staff, faculty expertise, and funding available for educational activities.

The McGaw Medical Center of Northwestern University is a consortium of five member hospitals and seven affiliated hospitals engaged in graduate medical education (GME) in Chicago, Illinois. McGaw sponsors 190 training programs and employs 1136 trainees. Northwestern Simulation™ is a state of the art simulation center that educates students, residents, fellows, and faculty at Northwestern University Feinberg School of Medicine. It is 13,000 square feet and contains high fidelity simulators, task trainers, operating suites, examination rooms, classrooms, and debriefing spaces. Northwestern Simulation’s™ faculty leaders have expertise in curriculum design, research, clinical skills assessment, and faculty development.

There was no central administration funding or oversight of SBME at the institutional level for GME simulation activities at McGaw before 2013. There were also no centralized faculty development workshops, mentoring, and structured curriculum development guidance materials available. Without adequate oversight, there was minimal concern for use of resources, and simulation programs were fragmented, operating in departmental silos. Faculty development and mentoring occurred only on an ad hoc basis.

Coincident with the recognition of increasing ACGME residency review committee requirements, the medical center allocated funds for academic year 2013–2014 GME simulation activities. These funds represented a small percentage of the overall GME educational budget, yet were contributed by all of the member institutions comprising our GME consortium. This new availability of funds required a more deliberate and transparent approach to allocation of simulation resources.

In 2012, the Northwestern Simulation™ faculty leaders were asked to develop a mechanism to distribute the approved institutional SBME funding for academic year 2013–2014. Simulation leaders were also asked to provide mentorship to create a larger institutional faculty cohort to sustain SBME over time. We developed a mechanism to submit and evaluate all GME-based SBME proposals at our institution in response to this request. The primary motivation for this dramatic change centered around the goal of promoting the development and implementation of high-quality educational sessions for healthcare providers. Our goals were to (a) establish a standardized mechanism for proposal submission and evaluation, (b) identify GME-based SBME curricula that would benefit from mentored improvement before implementation, and (c) ensure that funding decisions were fair and defensible. The intent was to develop a process that was grounded in sound educational principles, allowed for efficient administrative oversight, ensured approved courses were high quality, encouraged rigorous curriculum evaluation research, and provided opportunities for medical specialties that had not previously used SBME to receive mentoring and faculty development.

This article describes mechanisms created in 2012 at Northwestern University to review, evaluate, and make funding decisions about SBME curricula within GME programs. The proposal development process has evolved since initial implementation. The current description reports the processes used since spring 2015 to evaluate, improve, and fund proposed projects. We conclude with a discussion about challenges, successes, and limitations that informed this work.

## Proposal submission development

A multispecialty group of board-certified physicians (general and pediatric surgery, emergency medicine, anesthesiology, internal medicine, pediatrics), a simulation technician, a nurse educator, and administrative staff was convened in November 2012 to manage SBME proposal submission and review.

The first task was to create a curriculum proposal submission form using an iterative process. We designed the proposal structure to mirror the process by which curricula are developed. Medical curricula have been created using a variety of models ranging from subject centered to integrated and competency-based [6]. Specifically, our process followed the six-step approach to medical curricula development described by Kern and colleagues [7]. The six steps are:

1. Problem identification and general needs assessment;
2. Targeted needs assessment;
3. Goals and objectives;
4. Educational strategies;
5. Implementation; and,
6. Evaluation and feedback.

The Northwestern Simulation™ proposal submission form allows prospective users to describe the rationale for the proposed SBME program and provide an initial description of the event design and required resources (Table 1). The curriculum form requests basic information about the proposed SBME curriculum including, name, specialty, department, hospital affiliation, and contact information. Additional information also includes other funding sources for the proposed curricula (i.e., internal and external grants). Authors must provide a summary of relevant experience in medical education and SBME to determine if course developers or facilitators will need mentoring, including participation in faculty development workshops before starting an SBME program. Available workshops address skills such as (a) curriculum development, (b) creating simulation scenarios, and (c) effective debriefing for SBME.

**Table 1:** Components of Northwestern Simulation™ curriculum submission form

Primary Faculty and Teaching Faculty Information (including SBME experience)
Other sources of funding (if any)
Research Plan (including date of IRB approval)
Curriculum Description
Background/needs assessment
Learning objectives
Target learners
Location (simulation center, in-situ, classroom)
Brief summary of curriculum (including educational rationale, approach, plan for creating a safe learning environment, why SBME is proposed, and how this curriculum is relevant to and may impact clinical practice)
Assessment tools and plan
Relation of curriculum to ACGME requirements in your specialty
How your curricula will be evaluated for future quality improvement
References
Information for cost calculations
Number of sessions planned
Simulators required (tissue, high-fidelity mannequin, task trainer, virtual reality, standardized patient encounter)
Staffing needs
Room requests (bioskills spaces, debriefing, procedural training space, high fidelity simulation rooms, didactic/classroom)

*SBME* simulation-based medical education, *IRB* institutional review board, *ACGME* Accreditation Council for Graduate Medical Education

Authors address their *general problem and/or need* (Kern Step 1) and are asked to describe how the proposed curriculum fulfills an unmet need. Users must provide a rationale for the SBME curriculum supported by a needs analysis or justification. Prospective faculty provide information about why their simulation-based curriculum is an improvement over current educational methods. Authors explain how the proposal will meet specific *local needs* (Kern Step 2), with attention to how it might enhance or support existing curricula or fulfill specific ACGME requirements. Authors also must not exceed 2000 characters in this initial section to allow sufficient description of the activity while keeping the review process manageable. A brief list of relevant literature completes this section.

Next, authors must articulate learning *objectives* (Kern Step 3) for the session that align with the needs assessment. Online resources provide support to help authors craft learning objectives through web links to the “Writing Learning Objectives Guide” [8].

The next section of the form addresses session design where authors describe *educational strategies* (Kern’s Step 4) and their plans for *implementation* (Kern’s Step 5) of their SBME curricula. As all proposals use some form of simulation, here authors describe the specific type of simulation they will use and the space and equipment required for session implementation. Detailed simulation scenarios that are to be used during curriculum implementation should match with simulation modality and equipment needs.

We solicit specific details about resources required, including standardized patients, nurses, equipment, simulators, rooms, and video recording on the proposal submission form. The number and length of proposed sessions assists in determining appropriate resources. These data play a critical role in determining the feasibility, cost, personnel, space, and equipment needs for each proposed curriculum. Finally, proposal authors must read and acknowledge compliance with simulation center policies, such as ensuring a safe learning environment and adequate prescheduling time for simulation needs (e.g., standardized patients, nurses).

The final section of the curriculum proposal form requests information about how learners and sessions will be *evaluated* and receive *feedback* (Kern’s Step 6). Specifically, authors distinguish between sessions designed for formative or summative feedback and describe plans to ensure learner safety in terms of privacy and data confidentiality. Authors need to include a session evaluation form where learners provide feedback about simulation education sessions and instructors. We encourage measurement not only of immediate learner outcomes, but also potential downstream effects of SBME including improved patient care practices, safety, and patient outcomes. Our intent is that rigorous curriculum evaluation will produce scholarly products (e.g., presentations, publications) that contribute to the simulation research literature. However, this is not a current requirement for proposals to receive funding.

Previously funded proposals may be submitted for renewal. In addition to the above process for new curricula, renewal proposals must include an additional section to summarize learner outcomes and results of prior learner feedback. Authors need to describe how they are using this feedback for continuous quality improvement of the curriculum.

## Proposal review

The proposal review process has six steps:

1. Develop/revise the SBME proposal-scoring rubric
2. Establish a submission timeline
3. Create a review process for staff and faculty reviewers
4. Calculate proposal priority scores
5. Determine costs of proposed curricula
6. Make funding decisions

The same multispecialty group that developed the curriculum submission forms also created an SBME proposal scoring rubric. There were two goals when constructing the rubric. First, to rate proposals objectively to ensure that the highest quality submissions are funded and provide feedback to authors so curricula can be modified and improved. Second, to identify authors of innovative and clinically relevant proposals that may not receive fundable scores initially, but might benefit from faculty mentoring to improve proposals for resubmission and eventual funding.

We adopted a modified version of the US National Institutes of Health (NIH) grant proposal scoring as a foundation for our review system given the familiarity and acceptability among our curriculum authors and reviewers [9]. Scoring is performed on a nine point anchored scale [1 = exceptionally strong with essentially no weakness to 9 = very few strengths and numerous major weakness (Table 2)]. Proposals are scored in four categories: Significance, Approach, Impact, and an Overall score. *Significance* is judged based on proposal alignment with training program needs. Programs that link to ACGME milestones, justify that simulation is required by their subspecialty board, or explain how the curriculum addresses an educational need receive favorable scores in this category. *Approach* is scored based upon an assessment of whether the submitted curriculum can be implemented as proposed. Do the SBME curriculum’s objectives align with the implementation plan? Are the goals achievable in the time and space requested? Is the education sufficiently rigorous to provide benefit to learners or patients? Do proposed facilitators possess the expertise to deliver an SBME curriculum? *Impact* aims to judge if the proposed curriculum will exert a sustained benefit for learners and their patients. The Impact score was given additional anchors grounded in the four T’s of Translational Science as it relates to SBME [10–12]. T1 SBME outcomes measure the effects of simulation education in the laboratory, reflecting skill improvement demonstrated in the simulation environment. T2 SBME outcomes show improved patient care practices in the clinic or at the bedside. T3 SBME outcomes show improved patient outcomes such as reduction in complications. Finally, T4 SBME outcomes show collateral effects in ways that were not intended, or among learners that did not participate in the intervention (such as reduced healthcare costs or improved skills among other trainees). Using this framework, a proposal achieves a score of 1–3 for Impact only if the proposal demonstrates prior research showing T3 or T4 outcomes or intends to measure these outcomes within the project. Scores of 4–6 can be granted for T2 outcomes, and 7–9 for T1 outcomes. The *Overall* score is a rating for the entire curriculum. The Overall score allows reviewers to identify projects that need mentored improvement. Reviewers also provide specific comments for each section to justify their scores. All scores and comments are shared with proposal authors.

**Table 2:**
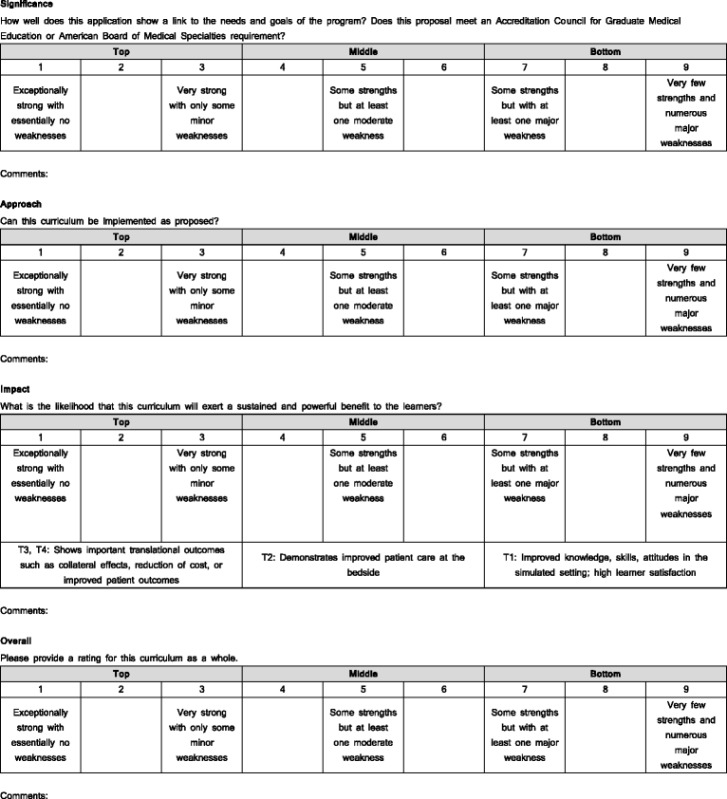
Curriculum Evaluation Form

ᅟ

**Fig. 1:**
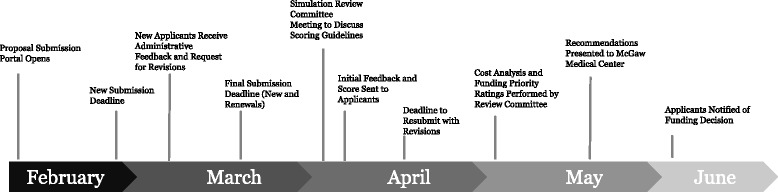
Graduate Medical Education Simulation Curriculum Proposal Processing Timeline for Academic Year 2015–2016. New submissions are those that did not receive funding in the previous year, while renewal applications are those that did

All Northwestern faculty members receive a *submission timeline* annually. From February to April, faculty may submit simulation proposals for funding consideration for the next academic year. Emails are sent to all program directors and department chairs announcing the submission process and deadlines. All faculty members are also invited to attend an annual lecture that describes the SBME curriculum proposal submission process. Deadlines for initial submissions and revisions are shared, as well as timelines for scoring and funding decisions. A sample submission timeline is given in the Fig. 1.

We created our *review process* to promote efficiency and fairness. First, administrative staff members screen all new proposals (that have not been submitted in previous years) for completeness, and to ensure instructions are followed. Proposals that are incomplete are returned to the author for editing and resubmission. Northwestern Simulation™ faculty and staff then review all new and renewal course proposals. All reviewers participate in a 1-h meeting to discuss and calibrate scores using the rubric, before individual SBME proposals are judged.

Each reviewer is assigned four to seven simulation course proposals to review individually depending on the total number of reviewers and proposals. Each reviewer spends from 1 to 3 h (renewal proposals require less total time than new proposals). Reviewers are initially assigned to review proposals by a simulation administrator and cannot review proposals from their own Department (medical specialty). In the first year of implementation, 10 reviewers reviewed 36 submissions. In the most recent cycle, seven reviewers scored 36 submissions. Reviewers represent a wide cross section of specialties (Emergency Medicine, Internal Medicine, Surgery, Anesthesiology, Pediatric Emergency Medicine, Medical Education) and roles (physicians, nurses, non-clinician curriculum experts) with numerous years of SBME course development experience (range 5–15 years).

Next, we *calculate proposal priority scores*. We use the formula: (Significance Score + Approach Score + Impact Score + 2 X Overall Score)/5 to derive the final proposal priority score. The mean of the two reviewers’ scores is used for new proposals and based on the scoring rubric, the most favorably rated proposals have overall scores closest to 1 with the least favorably rated proposals closer to 9. Proposal scores and reviewer comments are then returned to authors. Authors are invited to respond to reviewer comments, revise their proposals, and resubmit within two weeks. Revised proposals are re-evaluated by the original reviewer(s) and scores modified as appropriate.

The review committee meets to discuss the proposals after final scores are tabulated. The group discusses proposals where two reviewers’ scores are greater than 1 point apart and all proposals that receive scores 7 and higher (least favorable) or they deem need further discussion. The group adjusts scores based on consensus, similar to NIH study sections. The review committee also determines which proposals will have conditional funding. For instance, a new SBME curriculum might receive favorable scores for Significance, Impact, and Overall score. However, the approach may be weak due to lack of faculty with SBME experience. In such a case the score might be adjusted with conditional acceptance to ensure the author will work with simulation faculty and staff to improve the approach before project implementation. Some proposal authors may also need to participate in faculty development experiences before curriculum implementation. Finally, based on final scoring, a few proposals are not advanced for funding consideration.

Due to the complexity of simulation center usage, administrative personnel determine the costs for each proposal using predetermined institutional charges for various types of SBME. The final consensus review score and the subsequent cost analyses are then used to rank order all approved curricula. Proposals are also grouped by Department (medical specialty) to ensure that SBME curricula are accepted across the spectrum of GME programs. All favorably rated proposals are then reviewed to determine if options exist to more efficiently deliver an educationally rigorous curriculum at a lower cost. Final rankings are submitted to a steering committee of the McGaw Medical Center of Northwestern University to determine the funding line for proposals based on the available budget.

## Funding decisions to date

In 2013–2014, we received and funded all 29 proposals (mean score 2.88, SD 1.36). In 2014–2015, 38 proposals were submitted and 36 were funded (mean score 3.38, SD 1.24). In 2015-2016, 37 were submitted and 34 were funded (mean score 3.27, SD 1.35). Authors of curricula that do not receive funding receive specific feedback about their proposals and are invited to resubmit the next year. The two unfunded proposals in 2014–2015 were both successfully resubmitted and funded in 2015–2016. During the first 3 years of implementation, approximately 10% of proposals have required mentored improvement before implementation. However, all proposals receive mentoring as needed throughout the application process. In the first year, all accepted proposals were dominated by five departments. In subsequent years, over 15 departments submitted successful proposals.

## Discussion

This report describes the implementation of a rigorous SBME curriculum development, evaluation, and funding process for GME simulation activities guided by a defensible rationale. The ACGME has recently required residency programs to use competency-based Milestones to assess a resident’s achievement of competency during his/her progression through residency [13]. Simulation has been advocated as an effective means to assess trainee achievement of these competencies in a safer environment than actual patient care [14–17]. We know that traditional methods of training (vicarious learning) produce uneven skill acquisition [18–20]. Simulation training has advantages over traditional training that have shown to improve trainee skills [17, 19, 20], and reduce complications, and healthcare costs [14, 15, 21–26]. The demand for simulation-based resources in the coming years will increase as more healthcare training programs move toward adoption of simulation-based education and assessment.

## Program evaluation

Feedback about the approved proposals is currently obtained via several mechanisms to ensure ongoing program evaluation. First, simulation staff frequently bring concerns of deviation from approved curricular plans to Northwestern Simulation^TM^ leadership. Second, specific questions contained on renewal proposals ask authors to describe successes and challenges with implementation of their curricula, intended changes to the curricula for the renewal period, and a summary of learner evaluations. Third, simulation faculty and staff perform random audits by observing SBME curricula to ensure proposals are implemented as described in a safe and nurturing learning environment. Faculty (curricula authors) receive specific feedback about how they may improve their SBME curricula after these observations.

## Successes

The course proposal process we describe here has been successful for at least seven reasons. First, it provides a mechanism that allows identification and ranking of simulation-based courses that should receive funding based on a previously established NIH peer-review formula. This mechanism ensures that all simulation users have an opportunity to compete for limited space and funding. It also ensures that limited financial resources are used for the curricula that are well designed and valuable to learners and are aligned with the institutional mission. Second, faculty members that have limited simulation experience, and might have otherwise been excluded from SBME, are identified and mentored via faculty development programs for curriculum design, simulation scenario development, and debriefing. Third, the organized approach requires applicants to consider creation of SBME curricula in a deliberate fashion using Kern’s six-step curriculum design method [7]. Fourth, the transparent process reduces concerns about the allocation of limited GME funds. This is achieved through broad specialty representation, with multiple reviewers reaching consensus after group discussion, using a standardized and familiar scoring approach. Furthermore, all proposals submitted by inexperienced faculty or those with numerous years of simulation experience, are all evaluated using the same review system and discussed during the consensus meeting. Fifth, the curriculum development and submission process provides sufficient detail to estimate each project budget, allowing for alignment with institutional goals of patient safety, exposure to infrequent clinical presentations or procedures, and ACGME or specialty board requirements. Sixth, funding of proposals occurs annually. However, if an accepted and already funded curriculum acquires external grant funding during a fiscal year, the department can then use the grant money to cover SBME costs and use the center funding for new proposals in the same academic year. The new proposals must be vetted through the same curriculum review process. In fact, regardless of the source of funding, all GME-based SBME activities held at Northwestern Simulation™ are required to go through the curriculum review process. Institutional GME funding to support SBME is not a requirement of each application. Since implementation of our submission process, only one proposal has not requested institutional GME funding; their funding came from departmental sources. Finally, our model provides a clear understanding of what GME simulation use is planned over the course of the academic year allowing for effective scheduling across a large group of users.

## Challenges

The simulation curriculum process revealed several challenges that led to annual improvements. First, we needed to enhance communication to ensure all potential users were aware of the GME submission process and the availability of institutional funds. We used email and an annual lecture to publicize and describe the process to potential users. Despite these efforts, after the first year, we realized that some faculty members were still unaware of the simulation proposal and funding mechanism. An aggressive advertising campaign was launched, inviting hospital leadership and all faculty members to private tours and a simulation center open house (meet and greet). These efforts resulted in a 31% increase in SBME proposal submissions in year 2 of the process. Second, we revised the curriculum submission form each year to ensure it was “user friendly,” especially for relatively inexperienced SBME curriculum authors. Faculty development workshops were held, and internet links were embedded in the submission form to provide assistance developing learning objectives and to describe best practices for delivering feedback and formative and summative assessments. Third, the method of submission of a course proposal was modified over time. Proposals were completed in the first year as a Microsoft Word document and submitted via email. This method posed challenges in ensuring completion of all required sections for the proposal, as well as difficulties with organization and processing of the applications after receipt. A user-friendly, web-based form with pull-down menus and skip logic was needed. We introduced FluidReview (Fluidsurveys, Ottawa, ON, Canada), an online application system for grants, scholarships and awards in year three [27]. This system allows both submission and scoring of curriculum proposals. Individual user information can be entered to pre-populate subsequent submissions and draft proposals can be saved and edited before final submission. Fourth, we modified the scoring rubric each year. In year 2, we added more specific anchors and provided additional rater training before faculty reviewers graded proposals. Detailed anchors were added to the rubric to include translational science criteria for Impact score in year 3. Fifth, we realized more details were needed within SBME proposals to estimate costs accurately. We added several items addressing the type and duration of each simulation activity contained in the curriculum proposal, including specific types of simulators, equipment, rooms, and video needs. Finally, we clarified simulation lab use policies and procedures. During the first year we had many requests to add or reschedule sessions and to change simulation needs after funding was allocated. Authors must now submit changes to curriculum proposals in writing at least 1 month before implementation that must be approved by the review committee.

## Limitations

This proposal process describes an annual method for submission, review, and distribution of funding for GME SBME courses. Our proposal process has improved annually based on feedback. There are still limitations to the process which we also attempt to improve in each annual iteration. First, our procedure is based on an educational theory approach to curriculum design and not all health science centers have experience in SBME curriculum design. Second, successful implementation of our process also requires adequate faculty and staff resources. Faculty with SBME expertise are key to our approach to rating proposals and delivering faculty development courses and do so as part of their academic obligations to the medical school. We estimate approximately 0.35 full-time equivalent administrative support is needed yearly to oversee curriculum development, submission, and evaluation. Third, our faculty review process was designed to provide guidance for the distribution of institutional funding for GME simulation activities. A separate and distinct process is applied to non-GME activities (e.g., external users who are seeking space for in-situ systems testing, device testing, or clinical research and medical student or continuing medical education activities). Fourth, although several studies have shown downstream impact from SBME on patient care [14, 19, 21, 22], we do not have such expected outcomes from proposals funded under this mechanism. We are unable to determine if this new process resulted in increased academic output from the submitting faculty due to the short duration since implementation. Fifth, the majority of the submitted projects were funded. However, the purpose of our proposal process was to not only discriminate among the submitted curricula, but also improve curricula by giving all authors feedback, streamline the process, increase operational efficiencies, provide transparent justification for distribution of limited funds, and identify borderline proposals just below the funding cut-off which might be successful with additional mentoring. Finally, we acknowledge that other schools of health professions education may have limited personnel and financial resources which prevent such an ambitious curriculum review procedure. We encourage tailored adaptation of the model presented in this report to the needs and conditions that make sense in other educational settings. For example, healthcare simulation educators may rely solely on the needs assessment feature of the Kern curriculum development model to isolate educational gaps that warrant local attention. Administration and information technology resources can be limited by using simpler and cheaper applications to submit proposals (e.g., Microsoft Word). Additionally, for those centers considering implementing a similar process, a survey of SBME course directors might provide additional guidance regarding how to specifically adapt our process.

In conclusion, we developed a rigorous mechanism to guide faculty and simulation leadership with SBME curriculum submission, review, and funding decisions. Specifically, this mechanism allows faculty members to submit proposed SBME curricula for funding, establishes a mechanism for evaluation of proposals with a uniform rubric, and ensures that funding decisions are fair and defensible. This process is grounded in sound educational principles, allows for efficient administrative oversight, ensures approved courses are high quality, and provides opportunities for medical specialties that had not previously used SBME to receive mentoring and faculty development.

## Abbreviations

ACGME: Accreditation Council for Graduate Medical Education
GME: Graduate medical education
NIH: National Institutes of Health
SBME: Simulation-based medical education
